# Genetic Variation/Evolution and Differential Host Responses Resulting from In-Patient Adaptation of *Mycobacterium avium*

**DOI:** 10.1128/IAI.00323-18

**Published:** 2019-03-25

**Authors:** N. Kannan, Y.-P. Lai, M. Haug, M. K. Lilleness, S. S. Bakke, A. Marstad, H. Hov, T. Naustdal, J. E. Afset, T. R. Ioerger, T. H. Flo, M. Steigedal

**Affiliations:** a Centre of Molecular Inflammation Research (CEMIR), Norwegian University of Science and Technology (NTNU), Trondheim, Norway; b Department of Clinical and Molecular Medicine, Norwegian University of Science and Technology (NTNU), Trondheim, Norway; c Department of Computer Science and Engineering, Texas A&M University, College Station, Texas, USA; d Department of Infection, St. Olavs University Hospital, Trondheim, Norway; e Department of Pathology, St. Olavs University Hospital, Trondheim, Norway; f Levanger Hospital, Health Trust Nord-Trøndelag, Department of Internal Medicine, Levanger, Norway; g Department of Medical Microbiology, St. Olavs University Hospital, Trondheim, Norway; Weill Cornell Medical College

**Keywords:** *Mycobacterium avium*, intracellular pathogen, pathogenesis

## Abstract

Members of the Mycobacterium avium complex (MAC) are characterized as nontuberculosis mycobacteria and are pathogenic mainly in immunocompromised individuals. MAC strains show a wide genetic variability, and there is growing evidence suggesting that genetic differences may contribute to a varied immune response that may impact the infection outcome. The current study aimed to characterize the genomic changes within M. avium isolates collected from single patients over time and test the host immune responses to these clinical isolates. Pulsed-field gel electrophoresis and whole-genome sequencing were performed on 40 MAC isolates isolated from 15 patients at the Department of Medical Microbiology at St. Olavs Hospital in Trondheim, Norway. Isolates from patients (patients 4, 9, and 13) for whom more than two isolates were available were selected for further analysis. These isolates exhibited extensive sequence variation in the form of single-nucleotide polymorphisms (SNPs), suggesting that M. avium accumulates mutations at higher rates during persistent infections than other mycobacteria. Infection of murine macrophages and mice with sequential isolates from patients showed a tendency toward increased persistence and the downregulation of inflammatory cytokines by host-adapted M. avium strains. The study revealed the rapid genetic evolution of M. avium in chronically infected patients, accompanied by changes in the virulence properties of the sequential mycobacterial isolates.

## Abstract (precis)

Members of the Mycobacterium avium complex (MAC) are characterized as nontuberculosis mycobacteria and are pathogenic mainly in immunocompromised individuals. MAC strains show a wide genetic variability, and there is growing evidence suggesting that genetic differences may contribute to a varied immune response that may impact the infection outcome.

## INTRODUCTION

Members of the Mycobacterium avium complex (MAC) are a group of opportunistic pathogens consisting of M. avium and M. intracellulare species which primarily affect individuals with weakened immune systems (1–4). However, even healthy individuals can be infected by M. avium, and in healthy children, the disease manifests as lymphadenitis (5–7). M. avium infections are very hard to treat, and many antimycobacterial drugs fail to clear the infection even after prolonged treatment for 18 to 24 months (8, 9).

The host response to Mycobacterium tuberculosis has been extensively examined over the years. Infections with nontuberculous mycobacteria are less well characterized, although the focus has been increasing in recent years (10). M. avium lacks several of the key virulence factors of M. tuberculosis but can still establish chronic infections. Macrophages are central to the defense against mycobacterial infections, but they also end up hosting the pathogens, as the pathogens manipulate cell-autonomous host defenses. When infected, macrophages release interleukin-12 (IL-12), which aids in the activation of T cells, leading to interferon gamma (IFN-γ)-producing CD4^+^ T helper 1 (Th1) cells, thought to be essential in fighting mycobacterial infections (11). IFN-γ acts back on the macrophages and strengthens their antimicrobial capacities. Other factors, such as tumor necrosis factor alpha (TNF-α), produced by activated macrophages and T cells, are important not only in enhancing the microbicidal capacity but also in inducing the adaptive response in synergy with IFN-γ (12, 13). Proinflammatory cytokines like IL-6 and IL-1β have also been shown to play a role in mycobacterial infections (11, 14–17). IL-1β, along with IL-6 and TNF-α, has been observed to be suppressed by the most virulent strains of M. avium (18).

The genome of M. tuberculosis is thought to be relatively unaffected by the host environment (19), but data from macaques suggest that M. tuberculosis acquires mutations during long-term M. tuberculosis infection (20). Genomic analysis has revealed genomic heterogeneity in M. avium greater than that which can be seen in M. tuberculosis (21–23). This is probably a consequence of the variety of niches that M. avium species occupy (such as soil, freshwater, and showerheads), whereas M. tuberculosis is restricted to growth in human hosts and may have suffered a loss of genomic diversity among extant lineages worldwide through an evolutionarily recent population bottleneck event (24). Genetic change within an infected patient has not been investigated for M. avium infection. The effect of genetic variation on virulence and pathogenesis is even less well understood. An early study on M. avium virulence found that strains varied in virulence depending upon where they were isolated from, despite having a very similar genomic composition (25, 26). Genetic changes could be selected for when they provide increased virulence and persistence. We hypothesized that the hostile environment during chronic M. avium infections in humans would result in genetic changes in the infecting M. avium strain and possibly alter the virulence of the strain. In the present study, we investigated genetic changes by sequencing M. avium isolates sampled from individual patients over time and studied the host responses to these sequential isolates *in vitro* in primary mouse macrophages and *in vivo* in mice.

## RESULTS

### Sequential sampling from patients with MAC infection shows persistent infection over time.

**FIG 1:**
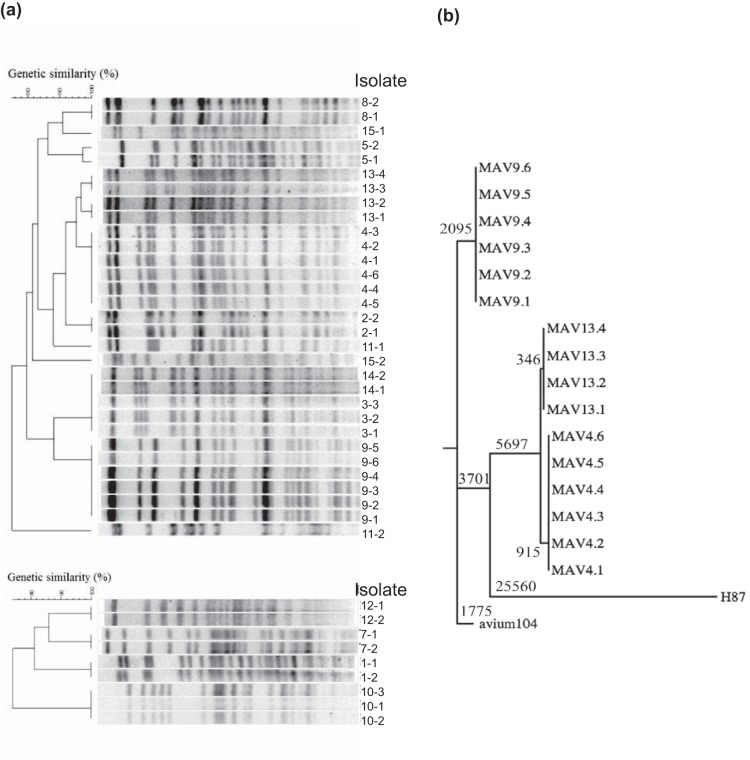
Genetic comparison/classification of clinical MAC isolates from patients. (a) Pulsed-field gel electrophoresis of 40 clinical isolates from 15 patients was performed using a SnaBI typing method. Restriction enzymatic digestion by SnaBI created distinct profiles that could be used to distinguish between isolates from different patients and between isolates taken at different time points from the same patient. Dendrograms were generated based on cluster analysis using the UPGMA method and the Dice similarity coefficient to assist in visualizing SnaBI pattern similarity. (b) Maximum parsimony tree showing the phylogenetic relationship among the clinical isolates and two M. avium reference strains, 104 and H87. The lengths at the branches indicate the number of changes (SNPs).

For this study, 15 patients diagnosed with MAC disease were monitored over a period of from 1 month to 3 years. At least two consecutive bacterial samples were isolated from all patients with at least a 1-month interval, providing a total of 40 isolates (see Table S1 in the supplemental material). To differentiate between the two MAC species (M. avium and M. intracellulare), the results of melting curve analyses of 16S rRNA from the 40 isolates were compared to those for type strains of M. tuberculosis, M. avium, and M. intracellulare (Fig. S1). Thirty-one isolates were identified as M. avium, and nine were identified as M. intracellulare. All isolates from each patient were identified to be the same species, suggesting either that the same bacterium persists over time or that the patient was reinfected with the same species. To evaluate whether strains isolated from a patient at different times represented persistent infection or reinfection with a new strain of the same species, we performed pulsed-field gel electrophoresis (PFGE) using SnaBI restriction analysis (Fig. 1a). Sequential isolates were found to be of indistinguishable genotypes in 13 of the 15 patients, suggesting persistent infection by a single bacterial strain. Phylogenetic cluster analyses based on PFGE profiles were separately carried out for each species (Fig. 1a). All M. avium and M. intracellulare isolates showed distinct and similar SnaBI profiles. For patients 4, 9, and 13, for whom more than two consecutive M. avium isolates were recovered from the same patient, we observed that most of the isolates had identical (patients 4 and 9) or very similar (patient 13) SnaBI profiles, suggesting the presence of a single strain persisting over time. For patient 13, the presence of an extra band in the DNA profile of isolates 13.3 and 13.4 indicated that a genetic change occurred sometime between the times of sampling of isolates 13.2 and 13.3.

### WGS reveals accumulation of SNPs and genetic variation in M. avium during persistent infection.

Genetic changes in some of the serial intrapatient isolates were observed from PFGE analysis. To further investigate genetic changes, whole-genome sequencing (WGS) of the isolates was performed. The genome sequences were analyzed for single-nucleotide polymorphisms (SNPs), using M. avium 104 as a reference strain, and a phylogenetic tree was constructed. We observed that the isolates from patient 9 were closely related to M. avium 104, whereas isolates from patients 4 and 13 had accumulated several thousand additional shared SNPs (relative to a genome size of ∼5.5 Mbp for M. avium), though they were still genetically closer to M. avium 104 than to other M. avium subsp. *hominissuis* strains, such as H87 (27) (Fig. 1b). The isolates from patients 4 and 13 also had several large-scale polymorphisms (including insertions with sizes of up to 22 kb and deletions with sizes of 40 kb), consistent with their higher diversity from M. avium 104. Overall, the genome sequences of intrapatient isolates were highly similar, with only 0 to 19 SNPs between any intrapatient pair (Table S3), strongly supporting the suggestion that each patient maintained a unique infecting strain.

**FIG 2:**
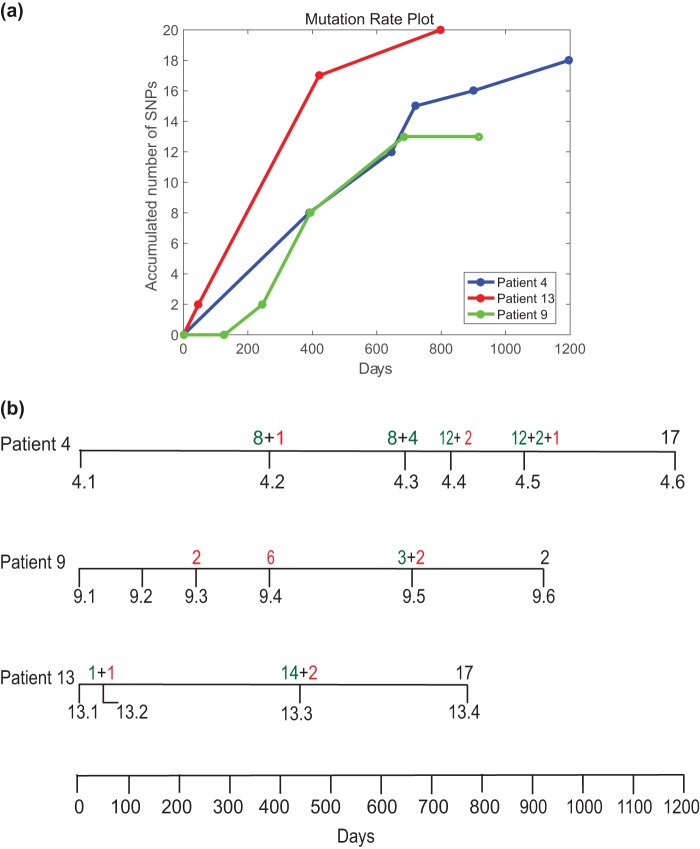
(a) Plot of cumulative number of unique SNPs observed from whole-genome sequencing of M. avium clinical isolates sampled over time from single patients. (b) Diagrammatic representation of the timeline of sample collection from patients 4, 9, and 13 and the corresponding SNP development. The numbers of SNPs in green represent fixed mutations, whereas the numbers in red are unique to that particular isolate and were lost in the subsequent isolates.

To establish the genetic variation between the intrapatient isolates of M. avium, we considered patients from whom more than two consecutive isolates were collected. Patients 4 and 9 provided six isolates, whereas patient 13 provided four isolates. Figure 2a shows the accumulation of novel SNPs over time in each patient. In patient 4, a comparison of later isolates to the first isolate, isolate 4.1, revealed that a total of 18 SNPs (listed in Table S2) accumulated over a period of 1,196 days. In addition, we observed that 14 of the SNPs were maintained in subsequent isolates, indicating a high degree of fixation of the mutations in the infecting strain (Fig. 2b). The accumulation of SNPs in isolates from patient 4 steadily increased with time, with 8 SNPs being observed at the second time point (isolate 4.2, 390 days), and 7 of these SNPs were maintained at all successive time points analyzed.

In contrast to the SNPs observed in isolates from patient 4, the SNPs observed in earlier isolates from patient 9 were mostly not maintained in subsequent isolates. Although a total of 12 SNPs was identified for the series of isolates, only 2 mutations were fixed in subsequent isolates. We observed no differences in preservation between synonymous and nonsynonymous mutations that could explain the difference in the level of fixation in subsequent isolates (Table S2). We speculate that these isolates could be derived from distinct lesions in the host lung, consistent with observations in M. tuberculosis infections that genetically distinct lineages can arise in different lesions and be maintained as micropopulations in the host lung, each accumulating a different set of SNPs over the course of infection (20). They could also represent heterogeneous subpopulations that spontaneously arise but that are subsequently lost due to a relative lack of fitness (28).

When isolate 13.1 was used as a reference for isolates from patient 13, the subsequent isolates exhibited an accumulation of SNPs similar to that seen in patient 4, with 19 SNPs in total being detected within 4 isolates over a period of 798 days (Fig. 2b). The majority of SNPs occurred over the first year between isolate 13.2 and isolate 13.3, where 14 SNPs occurred, of which 12 were conserved until the last isolate, isolate 13.4. However, when we compared all the isolates from patient 13 (including isolate 13.1) to M. avium 104 as an outgroup, we observed that some SNPs were associated with isolates 13.1 and 13.2 and others were associated with isolates 13.3 and 13.4 (Table S3). This pattern of SNPs among isolates from patient 13 relative to the sequence of M. avium 104 suggested that the four isolates represented two distinct lineages (shown as two lines for patient 13 in the plot of accumulated SNPs in Fig. S5). Supporting the hypothesis that these isolates represent two distinct lineages, we observed an additional band in the PFGE profile for isolates 13.3 and 13.4 compared to isolates 13.1 and 13.2 (Fig. 1a), In addition, sequencing revealed that a putative prophage (carrying MAV0799 to MAV0845) appeared to be deleted in isolates 13.1 and 13.2 but not in isolates 13.3 and 13.4. These observations together indicate the presence of 2 subtypes that may have originated within patient 13. Due to a lack of multiple samples at each time point, it is difficult to further substantiate this observation. A similar analysis was performed for patients 4 and 9. For patient 9, the SNPs did not change with respect to M. avium 104; however, for patient 4, 4 mutations now appeared to associate uniquely with isolate 4.1 and the rest appeared to associate with isolates 4.2 to 4.6 (Table S3). This suggests that there were 2 subtypes in patient 4 as well.

Based on the accumulated number of unique SNPs observed in each patient over time, the maximum likelihood (ML) Poisson estimates of the mutation rates among intrapatient isolates were calculated using the poissfit tool in Matlab. The ML estimates of the mutation rates were 5.25 (95% confidence interval [CI], 3.25 to 8.02), 3.76 (95% CI, 2.25 to 5.88), and 6.22 (95% CI, 4.02 to 9.19) SNPs per genome per year for patients 4, 9, and 13, respectively (determined using the data from Table S3). This results in estimated mutation rates of 0.967 × 10^−6^, 0.693 × 10^−6^, and 1.15 × 10^−6^ SNPs per site per year for patients 4, 9, and 13, respectively. To eliminate possible biases due to selection pressure resulting from drug treatment, SNPs in loci potentially related to drug resistance (listed in Table S6) were excluded from the calculation. Although mutation rates within a single infected patient have not been studied previously for M. avium infections, these rates are an order of magnitude higher than the mutation rates among clinical isolates from related species, such as M. tuberculosis (∼0.5 SNPs per genome per year) (29).

### Host adaptation can change the ability of M. avium to survive in murine bone marrow-derived macrophages (BMDMs).

**FIG 3:**
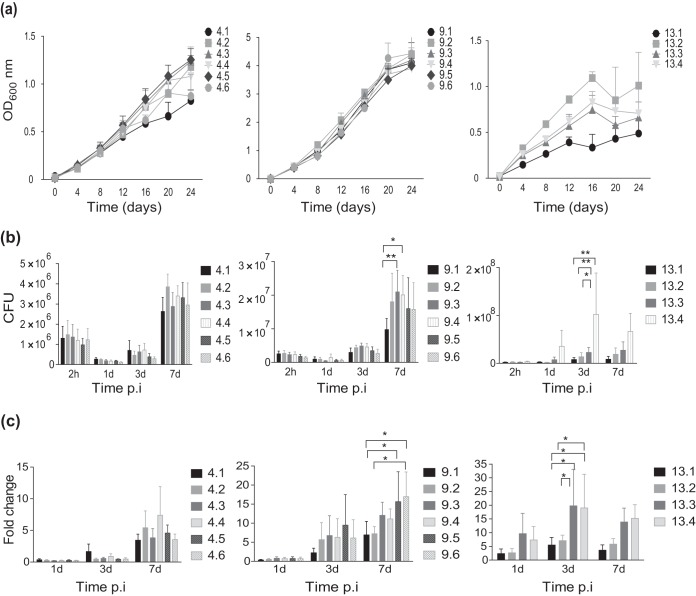
Growth in broth and intracellular replication in macrophages of sequential M. avium isolates. (a) The growth curves of sequential M. avium isolates from patients 4, 9, and 13 grown for a period of 24 days were recorded. Isolates are numbered chronologically from the time that they were collected from the patients. Recordings were performed in triplicate; the OD values show the mean ± SEM. (b) Intracellular replication in murine BMDMs infected with isolates from patients 4, 9, and 13 at an MOI of 10. CFU counts from lysed macrophages were determined at 1, 3, and 7 days after infection. (c) Intracellular bacterial counts represented as the fold change normalized to the uptake of bacteria at 2 h postexposure/postinfection. Bars represent the mean ± SEM from four independent experiments. *P* values were determined by repeated-measures two-way ANOVA with the Tukey posttest. *, *P* < 0.05; **, *P* < 0.01.

In order to evaluate whether changes within the isolates from patients 4, 9, and 13 were a response to host adaptation, we first studied the ability of these bacteria to grow in culture (Fig. 3a). Only minor differences in growth rates were observed for isolates from patients 4 and 9. However, for patient 13 we observed relative growth impairment for the first isolate compared to the growth of the other 3 isolates (Fig. 3a). Isolates were next compared for intracellular growth in murine macrophages over 7 days. Enumeration of bacteria (the number of CFU) at 2 h postinfection (p.i.) suggested equal uptake for all isolates (Fig. S2). For patients 9 and 13, a significant increase in the number of CFU between the first and the last isolates was observed (Fig. 3b and c). For patient 9, the number of CFU at day 7 increased 1.7-fold, from 9.7 × 10^6^ for isolate 9.1 to 1.6 × 10^7^ CFU for isolate 9.6, and for patient 13, the number of CFU increased 7.8-fold, from 8.5 × 10^6^ for isolate 13.1 to 6.6 × 10^7^ for isolate 13.4 from day 3 to day 7 p.i., suggesting that these strains increased their virulence during infection. For patient 4, no difference in the ability to survive inside macrophages was observed (2.6 × 10^6^ CFU for isolate 4.1 versus 3.0 × 10^6^ CFU for isolate 4.6). Taken together, our results suggest that M. avium dynamically adapts to the hostile environment of the host, thus facilitating persistence/chronic infection.

### Host adaptation can change the inflammatory properties of M. avium.

**FIG 4:**
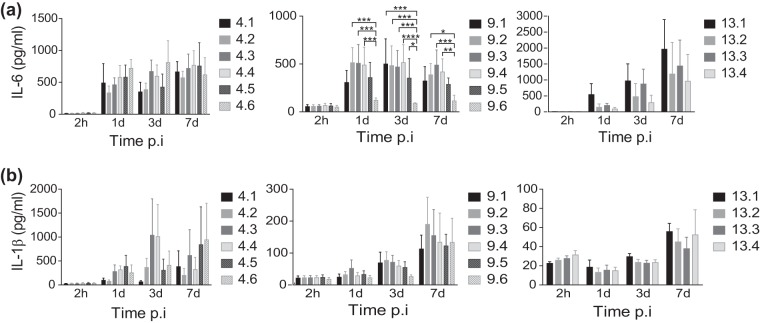
Downregulation of proinflammatory cytokines in M. avium-infected mouse macrophages. Murine BMDMs were infected with the sequential M. avium isolates from patients 4, 9, and 13 at an MOI of 10. The levels of IL-6 (a) and IL-1β (b) were measured from supernatants at 2 h, 1 day, 3 days, and 7 days postinfection (p.i.). Bars represent the mean ± SEM from three or four independent experiments. *P* values were determined by repeated-measures two-way ANOVA with the Tukey posttest. *, *P* < 0.05; **, *P* < 0.01; ***, *P* < 0.001; ****, *P* < 0.0001.

Inflammatory responses are central in controlling infection, and we and others have previously shown that the negative regulation of inflammatory responses by Keap1 or by depletion of Toll-like receptor (TLR) signaling facilitates the intracellular replication of M. avium in human primary macrophages (30–32). We therefore performed a broad systematic screen on the early expression of inflammatory genes to identify changes in immune stimulatory properties that could explain the change in survival. Murine BMDMs were infected with the first and the last clinical isolates of patient 9 at a multiplicity of infection (MOI) of 10 for 6 h. Genes that were differentially expressed (in which expression was induced or repressed at least 2-fold) between macrophages infected with the last isolate and those infected with the first isolate were identified and plotted (Fig. S3). The analysis revealed the downregulation of many proinflammatory cytokines, and we decided to measure the protein levels of two of them, IL-6 and IL-1β, from the supernatants of BMDMs infected with all isolates from patients 4, 9, and 13 (Fig. 4a and b). IL-1β secretion was low and highly variable for all strains, whereas IL-6 secretion was more potently induced. As the gene expression data indicated, we observed that despite an increased bacterial load in the macrophages infected with the final isolates of patients 9 and 13, the IL-6 levels were lower in the supernatants of macrophages infected with the final isolates than in the supernatants of macrophages infected with the first isolates. These results are in line with those of previous studies (33), and the reduced inflammation may facilitate intracellular survival (Fig. 3). However, patient 4 isolates did not show a similar reduction in cytokine production, which confirms the level of variation that has been previously reported (31).

### Host adaptation can change the ability of M. avium to survive in mice.

**FIG 5:**
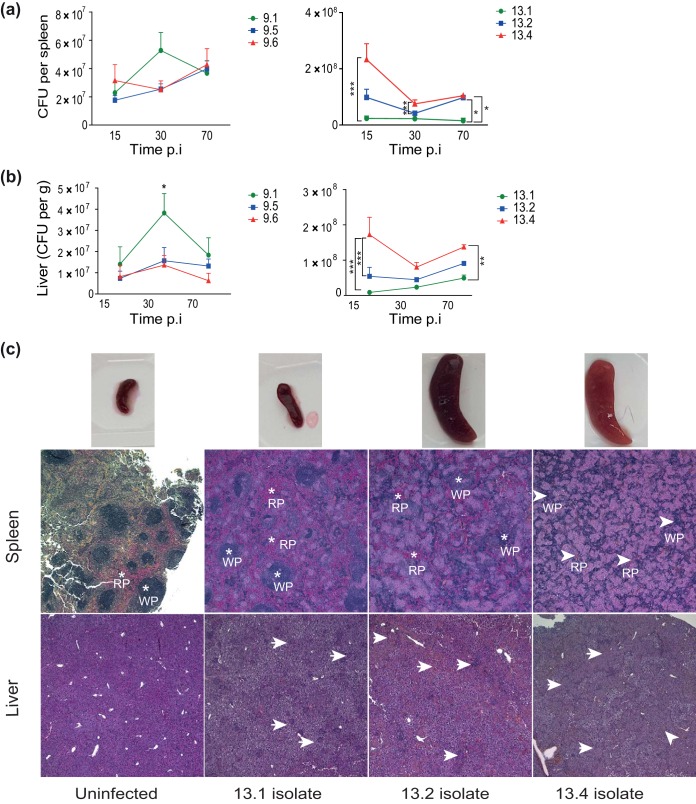
Mycobacterial load and tissue pathology in mice infected with sequential M. avium clinical isolates. (a and b) C57BL/6 mice were infected intraperitoneally with M. avium isolates from patient 9 (isolates 9.1, 9.5, and 9.6) and patient 13 (isolates 13.1, 13.2, and 13.4) for 15, 30, and 70 days. Bacterial loads in spleen and liver tissue are shown as the mean number of CFU per spleen ± SEM and the number of CFU per gram ± SEM for liver from two experiments with four mice in each group, respectively. *P* values were determined by repeated-measures two-way ANOVA and Bonferroni’s posttest. *, *P* < 0.05; **, *P* < 0.01; ***, *P* < 0.001. (c) Histological examination of spleen and liver from mice infected with M. avium isolates 13.1, 13.2, and 13.4 at day 70 postinfection. (Top) Spleens; (middle) spleen tissue histology sections (RP, red pulp areas; WP, white pulp areas); (bottom) liver histology. Arrowheads point to granulomatous structures. Images were taken at ×40 magnification. Experiments were done twice with similar results each time; the findings from one representative experiment are shown.

To validate if *in vitro* observations were reflected *in vivo*, we infected C57BL/6 mice with isolates from patients 9 (isolates 9.1, 9.5, and 9.6) and 13 (isolates 13.1, 13.2, and 13.4) (Fig. 5). We chose to investigate isolates from patients 9 and 13, as the *in vitro* data strongly indicated that the last isolates from these patients showed increased survival within macrophages compared to the early isolates. At days 15, 30, and 70 of infection, we measured the number of bacteria from both the spleen and liver of mice (Fig. 5a and b, respectively). Overall, the bacterial loads were constant or increased slightly over time for most isolates in both the liver and spleen.

In mice infected with strains from patient 9, the only significant difference was seen in the liver at day 30 p.i., where there was an increase in the number of CFU of the first isolate compared to the number of CFU of the last isolates (isolates 9.5 and 9.6) (*P* < 0.05). This is incongruent with our observations in macrophage infections (Fig. 3b and c). However, the *in vivo* data suggested no overall difference in virulence between the three isolates from patient 9 (Fig. 5a and b, left). For patient 13, the *in vivo* condition reflected our observations of increased infectivity that we made in macrophages (Fig. 3b and c). Throughout infection, we found significantly increased bacterial loads in liver and spleen from mice infected with the last isolate, isolate 13.4, compared to those in liver and spleen from mice infected with isolates 13.1 and 13.2 (Fig. 5a and b, right), suggesting a gain in virulence over time. At day 70, the spleen size increased substantially in mice infected with isolates 13.2 and 13.4 compared to that in mice infected with isolate 13.1, and the color of the spleens from mice infected with isolate 13.4 was strikingly paler than that of the other spleens (Fig. 5c, top row).

On further histological examination of the spleens on day 70 p.i. (Fig. 5c, middle row), we observed that the structure of the spleens of mice infected with isolate 13.4 was almost replaced by coalescing granulomas. In contrast, smaller amounts of single small or medium-sized granulomas, in part coalescing for isolate 13.2-infected spleens, were present in isolate 13.1- and 13.2-infected spleens on day 70 p.i. Myeloid precursor cells and megakaryocytes were found in all affected spleens, indicating extramedullary hematopoiesis. A similar pattern of granulomatous infiltration was seen in liver sections on day 70 p.i. (Fig. 5c, bottom row); however, the distinction between normal tissue and granulomas was less obvious in the liver than in the spleen. Infection with the 13.4 isolate resulted in replacement of much of the normal liver tissue with sheets of granulomas. The granulomas were sparse and mostly separated for isolate 13.1-infected liver, and the granulomas were moderate in number for isolate 13.2-infected liver but started to coalesce. It is important to note that though isolate 13.1 showed impaired growth *in vitro*, *in vivo* it still managed to survive, albeit poorly compared to the survival of isolate 13.4. Taken together our data suggest that the M. avium isolates recovered from patient 13 changed properties over the course of infection in the patient, which increased the ability of the organism to survive. Finally, we investigated the cytokine levels in organ homogenates from infected mice. The decrease in the levels of IL-6 and IL-1β observed in macrophages could not be observed in liver homogenates, in which the levels were steady over time and between isolates (Fig. S4).

### Splenic CD4^+^ and CD8^+^ effector cytokines decrease over time in mice infected by all M. avium isolates from patients 9 and 13.

**FIG 6:**
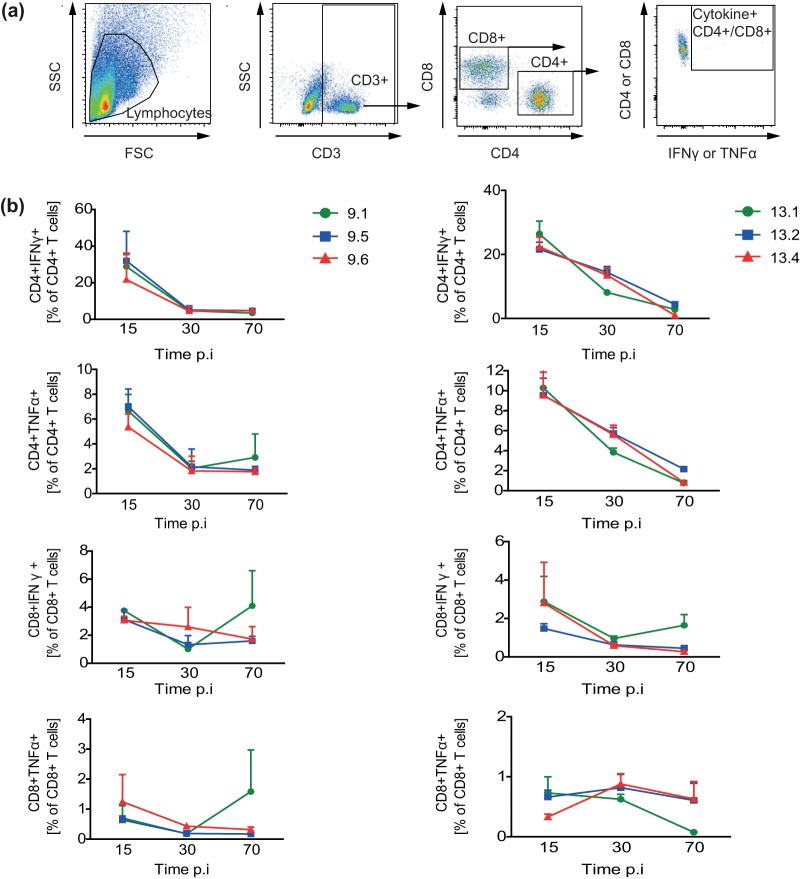
M. avium-specific splenic T cell responses. C57BL/6 mice were infected with isolates from patient 9 (isolates 9.1, 9.5, and 9.6; left in panel b) and patient 13 (isolates 13.1, 13.2, and 13.4; right in panel b) for 15, 30, and 70 days. M. avium-specific T cell responses were measured from *in vitro* M. avium restimulation of splenocytes at 15, 30, and 70 days postinfection (p.i.). IFN-γ and TNF-α production from CD4^+^ and CD8^+^ T cells was analyzed by intracellular flow cytometry. (a) Gating strategy. SSC, side scatter; FSC, forward scatter. (b) Results are represented as the percentage of T cells producing the indicated cytokine. Results show the mean ± SEM from two experiments with four mice analyzed in each group.

It has previously been demonstrated that M. avium infections elicit a CD4^+^ Th1 immune response, which is associated with resisting the spread of the pathogen (34, 35). In our study, we analyzed M. avium-specific IFN-γ or TNF-α (effector cytokine) production from CD4^+^ and CD8^+^ T cells that were isolated from the spleens of infected mice (Fig. 6). We observed that the frequencies of both M. avium-specific CD4^+^ and CD8^+^ T cells were high on day 15 p.i. and rapidly decreased by day 30 for patient 9 and decreased more gradually to day 70 p.i. for patient 13. This decrease in M. avium-specific T cells was observed with all tested M. avium isolates, despite sustained or even increased tissue bacterial loads (Fig. 5). The frequency of cytokine-producing CD8^+^ T cells was lower (<5%) than that of CD4^+^ T cells (20% to 40%) and did not change much over the course of infection.

## DISCUSSION

In the current study, we evaluated the mutations acquired by M. avium during persistent infection in human patients and further tested the pathogenesis of these isolates in macrophages and mice. Within macrophages, the M. avium isolates from patients 9 and 13 exhibited higher bacterial loads and downregulated inflammatory cytokines. However, the last isolates from patient 13, but not patient 9, showed increased survival in mice compared to the first isolate.

Most intrapatient isolates showed similar PFGE profiles, suggesting persistent infection with a single MAC strain. On WGS analysis, we found variable mutation rates in sequential intrapatient isolates. If we compare strains from different patients, our findings are in congruence with those of previous studies, which have reported high genetic diversity between M. avium isolates (36–38). The mutation rate observed for isolates within a patient ranged from 4.5 to 7.1 SNPs per year, which is significantly higher than the mutation rate of ∼0.5 SNP per year reported for M. tuberculosis isolates from humans (29) and macaques (20, 39). These observations further indicate that the genome of M. avium is more malleable than that of M. tuberculosis and more prone to variation, maybe due to an influence from host factors. In addition, we analyzed the patient isolates for SNPs in two ways. The first was to use the sequence of the first isolate from each patient as the founder strain and tabulate the SNPs present in the subsequent isolates (see Table S2 in the supplemental material). For the second analysis, all isolates from patients were treated as independent cultures and compared to the reference strain, M. avium 104, as an outgroup. For patients 4 and 13, in the second analysis, two subclonal populations were uncovered (Table S3). These could have been due to either the microevolution of a single founder strain in different lesions or mixed infection/reinfection. The plausibility of the former is more likely than that of the latter, as the PFGE patterns were nearly identical and the WGS patterns of the differences found within sequential isolates were identical to each other. The likelihood of being reinfected with such a genetically similar strain is low, based on a previous finding describing a high degree of genetic diversity in clinical and environmental isolates (40). In the case of patients 4 and 13, it can be speculated that the evolution of the isolates may have been linear or divergent. For patient 9, the predominance of unique SNPs in individual isolates that were not propagated to other isolates over time (i.e., not fixed) argues against linear evolution and instead supports the heterogeneity of the population structure *in vivo*. Studies in M. tuberculosis have examined the presence of subclones and intrapatient microevolution (41–44) and support the presence of divergent populations, which can be generated due to either antibiotics (42) or clinical severity (44). Thus, our analysis supports two hypotheses, linear and divergent microevolution.

Innate immune cells like macrophages are the first line of defense against mycobacterial infections. In M. avium pathogenesis, as in M. tuberculosis pathogenesis, macrophages are the primary host cells that initialize the containment of the bacterium (14, 30). We tested intracellular growth within murine macrophages and observed that though all isolates managed to survive within the macrophage, the last isolates collected from two patients (patients 9 and 13) survived better than the initial isolates from the same patients. This increased survival of certain isolates could be due to either their increased multiplication rate or the impaired ability of the macrophages to eradicate the bug (5, 25, 45, 46). In our experiments, we measured reduced levels of proinflammatory cytokines like IL-6 and IL-1β when macrophages were infected with later isolates than when they were infected with the initial isolate from a patient. The levels of IL-1β were highly variable in our experiments. Efficient IL-1β production requires that mycobacteria come into contact with the cytosol, and since M. avium is not present in the cytosol (32), this could explain the various results. The ability of M. avium to activate host defenses has previously been shown to vary between M. avium isolates, and the downregulation of proinflammatory cytokines is believed to promote survival of the bacterium (12, 18, 47). The present study is the first to indicate that the ability of M. avium to activate host defenses can change over the course of an infection. To support our *in vitro* observations, mice were infected with isolates from two of the patients, and infection showed a trend that was the same as that for the *in vitro* experiments for one set of isolates. It has previously been observed that there is a discordance between *in vitro* and *in vivo* data (5, 25). One explanation could be that *in vitro* macrophage infections represent an isolated system, as opposed to mice, where other immune cells, including the adaptive arm of the immune system, play an active role in curtailing the infection.

Cytokines like IFN-γ and TNF-α, produced by effector T cells, contribute to control of the infection at chronic stages (34). We previously demonstrated that in mice infected with M. avium 104, the induction of effector T cell responses coincided with a decrease in bacterial loads (35). In our current experiments, all the clinical isolates from both patients 9 and 13 impaired CD4^+^ effector T cell responses from about day 30 p.i. and exhibited bacterial persistence over time. Although *in vitro* there was an inverse relationship between the bacterial count and the inflammatory response, *in vivo* the increase in bacterial counts could not be explained by the production of inflammatory cytokines. We speculate that there could be other cells or effector molecules that play a role and that have not been studied, such as B cells, γ/δ T cells, neutrophils, and natural killer cells and effector molecules like IL-10, IL-17, and transforming growth factor β, all of which have previously been reported to have an important role in host defenses toward mycobacteria (48–52). Furthermore, the increased virulence exhibited by the isolates could be due to the high bacterial load. Studies have illustrated that at chronic stages of infection, due to prolonged exposure to high doses of antigen, T cells may undergo terminal differentiation and in parallel undergo apoptosis (53, 54). Considering the results obtained, we speculate that a diverse milieu can lead to modifications in the immune-modulating properties and intracellular proliferation of intrapatient isolates over time. Looking at WGS data (Table S2 and S3) for isolates from patient 13, isolate 13.4, in which an SNP in MAV0182 (K38T) was observed, exhibited a distinct increase in survival *in vivo* after the first time point. MAV0182 is annotated as a superoxide dismutase (SOD; Fe-Mn); SODs catalyze the dismutation of the superoxide radical to H_2_O_2_ and oxygen. In M. paratuberculosis and M. tuberculosis, SOD is actively secreted and has been shown to generate protective cellular immunity (55, 56). We speculate a similar role for MAV0182 and that the SNP may have aided in the survival of isolate 13.4. In addition, in isolate 13.2, which also exhibited increased growth, the SNP F267V in MAV2838 was observed. In fact, this was the only novel polymorphism observed in isolate 13.2 compared to the sequence of isolate 13.1. MAV2838 is an orthologue of the *oxyR* transcription factor, which is known to function during the oxidative response (57, 58). It is conceivable that the mutation F267V in MAV2838 confers a growth advantage through an adaptive response to oxidative stress. Further evaluation of the phenotypic effects of these SNPs, such as by recombineering, will be required to test their role in survival.

This is the first study analyzing the within-patient evolution of M. avium infection in humans. A possible limitation is that the cohort studied was small. In addition, only a single isolate was collected and analyzed at each time point. The analysis of multiple isolates from each time point would provide more statistical confidence to our findings and could give more evidence for evolution within the patient. In conclusion, we identified the intrapatient genetic variation of M. avium during persistent human infection and observed surprisingly high mutation rates. In addition, the response of the immune system to these clinical isolates was tested in macrophages and mice, demonstrating a highly adaptive microbe within individual patients over time. Further investigation of the correlation between SNPs and the adaptation of M. avium may provide insight into the multiple strategies which M. avium employs to resist chemotherapy and thereby help to develop more effective treatment strategies.

## MATERIALS AND METHODS

### Clinical isolates.

Forty clinical isolates from 15 patients diagnosed with MAC infections (2 to 6 consecutive isolates for each patient) were obtained from the Department of Medical Microbiology at St. Olavs Hospital in Trondheim, Norway. The bacterial burden within each patient varied from low to high, based on microscopy detection of mycobacteria (see Table S4 in the supplemental material). All isolates were previously characterized as MAC by the Norwegian Institute of Public Health in Oslo, Norway. The Regional Committee for Medical and Health Research Ethics approved this study (REK nord 2013/802).

The sixteen M. avium isolates investigated in depth in the current study were collected in chronological order (2005 to 2007) from the sputum of patients. The isolates were grown on 7H10 Middlebrook (Difco/Becton, Dickinson) medium supplemented with 10% albumin-dextrose-catalase (ADC; Difco/Becton, Dickinson). Single colonies were transferred to 7H9 Middlebrook (Difco/Becton, Dickinson) liquid medium supplemented with 10% ADC (Difco/Becton, Dickinson). All cultures were grown to the logarithmic phase, which was an optical density at 600 nm (OD_600_) of 0.5 to 0.6; bacterial cultures were pelleted down and resuspended in phosphate-buffered saline (PBS), followed by sonication, and were finally passed through a syringe to obtain a single-cell suspension. These suspensions were further used in either *in vitro* or *in vivo* infections.

### PFGE.

A modified pulsed-field gel electrophoresis (PFGE) protocol by Stevenson et al. (59) was employed. PFGE patterns were examined both by visual inspection and by computer-assisted analysis. Cluster analysis to compare SnaBI profiles and to construct dendrograms was performed using the Dice similarity coefficient and the unweighted pair group method with arithmetic means (UPGMA) in BioNumerics (v6.6) software (Applied Maths, Sint-Martens-Latem, Belgium). General guidelines for interpreting chromosomal DNA restriction patterns were used in evaluating the relatedness of the clinical isolates (60).

### Genome sequencing and assembly.

DNA was extracted by the cetyltrimethylammonium bromide-lysozyme method (61). Samples were sequenced on an Illumina HiSeq 2500 sequencer with a read length of 106 bp or an Illumina HiSeq 4000 sequencer with a read length of 150 bp; both sequencers were used in paired-end mode. The mean depth of coverage was 109.0 times (range, 51 to 158 times). Genome sequences were assembled by a comparative assembly method (62). The reads were mapped to the sequence of M. avium 104 (GenBank accession number NC_008595.1) as a reference genome using the Burrows-Wheeler algorithm (63). Then, regions with indels or clusters of single-nucleotide polymorphisms (SNPs) were identified and repaired by building local contigs from overlapping reads spanning such regions. Genome sequences were aligned using MUMmer (v3.20) software (64). SNPs were extracted according to the following criteria: coverage of ≥10 times (covered by at least 10 reads) with a purity of ≥70% (conversion to the nonreference nucleotide). SNPs in repetitive regions were filtered out (repetitive regions were defined as sites for which an overlapping 35-bp window matched the sequence elsewhere in the genome with at most 2 mismatches). A phylogenetic tree based on a genome-wide set of SNPs was constructed by maximum parsimony using the dnapars tool in the Phylip (v3.66) package of programs (65). Twelve randomly selected SNPs were confirmed using Sanger sequencing with the primers listed in Table S5.

### Macrophage culture and infection.

Bone marrow-derived macrophages (BMDMs) were generated by culturing bone marrow cells of C57BL/6 mice in RPMI 1640 medium (Sigma)–10% fetal calf serum (Gibco) and 20% L929 cell line supernatant for 4 days. Macrophages were seeded at 50,000 cells/well in a 96-well plate and infected with the M. avium clinical isolates at an MOI of 10 for 2 h. Cells were washed with Hanks balanced salt solution to eliminate extracellular bacteria. Three wells were lysed with PBS containing 0.02% Triton X-100 (Sigma), and the contents of the wells were plated in serial dilutions on 7H10 Middlebrook plates in triplicate to enumerate uptake by counting the number of CFU (time zero). At days 1, 3, and 7, infected cells were lysed and serial dilutions were plated on 7H10 Middlebrook plates in triplicate, to record the course of infection.

### Mouse infection experiments.

All protocols on animal work were approved by the Norwegian National Animal Research Authorities and carried out in accordance with Norwegian and European regulations and guidelines. C57BL/6 mice were bred in-house and used in the experiments at 6 to 8 weeks of age. Infection was performed by intraperitoneal injection of log-phase mycobacteria. The inocula injected for isolates from patient 9 were as follows: 5 × 10^7^ CFU/mouse for isolate 9.1, 9.3 × 10^7^ CFU/mouse for isolate 9.5, and 5.4 × 10^7^ CFU/mouse for isolate 9.6 for biological replicate 1 (BR-1) and 9.8 × 10^8^ CFU/mouse for isolate 9.1, 1.2 × 10^9^ CFU/mouse for isolate 9.5, and 1.1 × 10^9^ CFU/mouse for isolate 9.6 for BR-2. The inocula injected for isolates from patient 13 were as follows: 6.87 × 10^8^ CFU/mouse for isolate 13.1, 1.17 × 10^9^ CFU/mouse for isolate 13.2, and 1 × 10^9^ CFU/mouse for isolate 13.4 for BR-1 and 3 × 10^8^ CFU/mouse for isolate 13.1, 1.2 × 10^8^ CFU/mouse for isolate 13.2, and 1.1 × 10^8^ CFU/mouse for isolate 13.4 for BR-2. All inocula were administered in 0.2 ml PBS. The inoculum was measured by plating for determination of the number of CFU. At given time points after infection, mice were killed and the spleen and liver were collected. The bacterial load was measured by plating serial dilutions of organ homogenates (spleen, liver) on 7H10 Middlebrook plates.

### M. avium-specific T cell cytokine production.

Splenocytes were isolated and prepared for flow cytometry as described previously (35). Splenocytes were stimulated overnight with clinical M. avium isolates from patients 9 and 13 at an MOI of 1. Concanavalin A (2.5 μg/ml; Sigma) stimulation was used as a positive control, and unstimulated cells served as a negative control. A protein transport inhibitor (eBioscience) was added during the last 4 h of stimulation. Surface antigens were stained with monoclonal antibodies against CD3 (fluorescein isothiocyanate [FITC; BioLegend]), CD4 (Brilliant Violet 605 [BioLegend]), and CD8 (Brilliant Violet 785 [BioLegend]). After fixation (2% paraformaldehyde; Sigma) and permeabilization (0.5% saponin; Sigma), intracellular cytokine staining was performed for IFN-γ (phycoerythrin [BioLegend]) and TNF-α (allophycocyanin [BioLegend]). Flow cytometry was performed on a BD LSR II flow cytometer (BD Biosciences), and data were analyzed using FlowJo (FlowJo, LLC) and GraphPad Prism (GraphPad Software, Inc.) software.

### Histopathology.

Organ tissue samples were fixed in buffered formalin, processed through standard dehydration and clearing, placed in paraffin overnight, cut into 5-μm-thick sections, and stained with hematoxylin and eosin. Microscopic images were taken with a Lumenera Infinity 2 camera and INFINITY ANALYZE software (release 6.2; Lumenera Corp.) using a Nikon eclipse Ci microscope (Nikon) with a ×40 magnification.

### Statistics.

All values from intracellular replication experiments are the means from four independent experiments, performed in duplicate or triplicate. Results are presented as mean ± standard error of the mean (SEM) and were analyzed by two-way analysis of variance (ANOVA) followed by the Tukey posttest. The values obtained in the mouse experiments show the mean ± SEM from two independent experiments with four mice in each group. The results were analyzed by two-way ANOVA followed by the Bonferroni posttest. In all analyses, a *P* value of <0.05 was considered statistically signiﬁcant. Data analysis and statistical tests were performed with GraphPad Prism (v5.0) software (GraphPad Software, Inc.).

### Accession number(s).

The sequence data for the clinical isolates have been uploaded to the NCBI Sequence Read Archive under accession number SRP136774 and are available through the following links: https://www.ncbi.nlm.nih.gov/Traces/study/?acc=SRP136774 and https://www.ncbi.nlm.nih.gov/bioproject/?term=PRJNA447977.

## Supplementary Material

Supplemental file 1
