# Supplementary material for: Genetic Variation/Evolution and Differential Host Responses Resulting from In-Patient Adaptation of Mycobacterium avium
Source: Infect Immun. 2019 Mar 25;87(4):e00323-18. doi: 10.1128/IAI.00323-18 (PMC6434124; doi:10.1128/IAI.00323-18)
Supplement: Supplemental file 1 [file IAI.00323-18-s0001.pdf]

Supplementary Figure and Table Legends

FIG S1 Differential melting curves from 16S rRNA gene qRT-PCR. The curves of 19 clinical MAC isolates as well as positive (*M. intracellulare* and Mtb) and negative controls are shown.

FIG S2 Bone marrow derived murine macrophages were infected with clinical stains, collected in chronological order, at a MOI of 10. After two hours of infection, cells were washed and lysed to analyze uptake. Uptake is represented as log CFU and designated as zero hour.

FIG S3 Nanostring data. Murine BMDMs were infected with the first and last clinical isolate of Mav (9.1 and 9.6) at a MOI of 10. After 6 hours post infection, cells were lysed in RLT buffer and subsequently RNA was extracted to perform nanostring analysis. nCounter® GX Mouse Inflammation Kit was used and the results were analyzed on *Partek® Genomics Suite®* software.

FIG S4 Proinflammatory cytokines IL-6 (A) and IL-1β (B) measured from liver homogenates. Results are presented as ng cytokine per gram tissue.

FIG S5 Accumulated number of SNPs, accounting for sub clones. SNPs were counted relative to Mav 104 for patients 9 and 13. The isolates from patient 13 were treated as two separate lineages (13.1-2 and 13.3-4). For patient 4, SNPs in isolates 4.2-4.6 were counted relative to 4.2.

Table S1 Medical history and time line of sample collection for all the patients in the study.

Table S2 List of genes containing SNPs. Both synonymous and non-synonymous mutations were observed. Putative gene function mentioned for non-synonymous mutations. Mav 104 reference from KEGG database was used to annotate function.

Table S3 List of genes containing mutations (relative to Mav104, as an outgroup). Both, synonymous and non-synonymous mutations were observed. Putative gene function mentioned for non-synonymous mutations. Mav 104 reference from KEGG database was used to annotate function. The mutations for patient 13 are highlighted in red and blue to emphasize the two sub-series of isolates, (13.1, and 13.2) and (13.3, and13.4). The mutations for patient 4 are highlighted in red and blue to emphasize the two sub-series of isolates (4.1) and (4.2-4.6)

Table S4 Description of mycobacterial isolates; Specimen type, Specimen location, microscopy quantification and concentration of bacteria

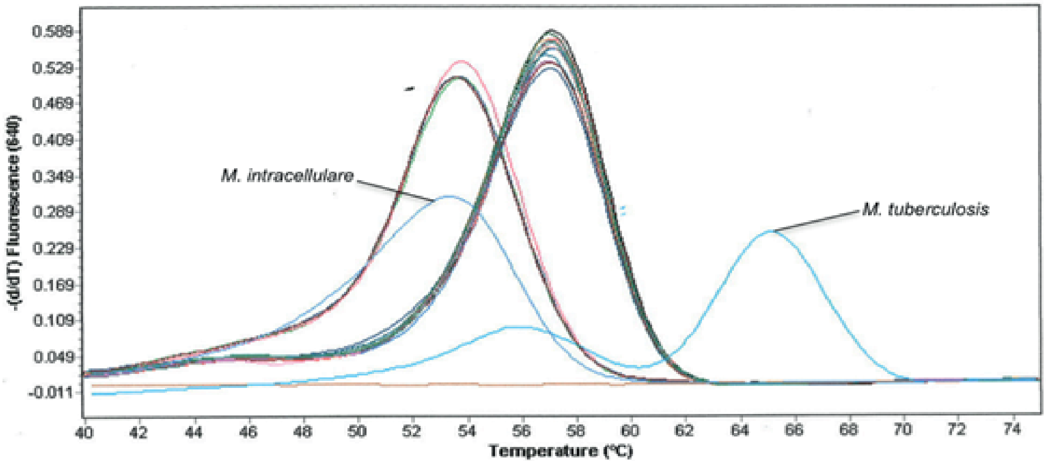

FIG S1 Differential melting curves from 16S rRNA gene qRT-PCR. The curves of 19 clinical MAC isolates as well as positive (*M. intracellulare* and Mtb) and negative controls are shown.

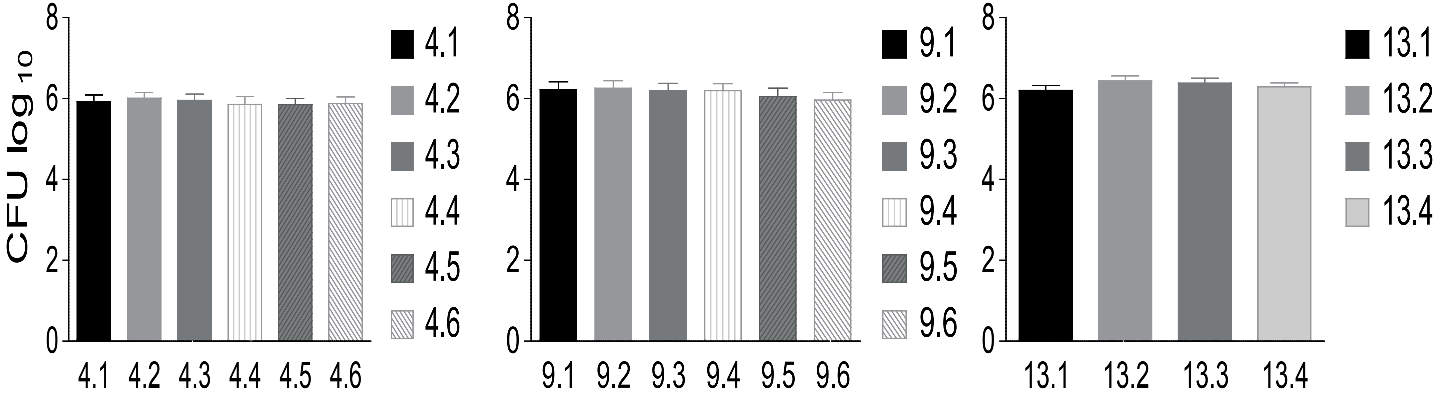

FIG S2 Bone marrow derived murine macrophages were infected with clinical stains, collected in chronological order, at a MOI of 10. After two hours of infection, cells were washed and lysed to analyze uptake. Uptake is represented as log CFU and designated as zero hour.

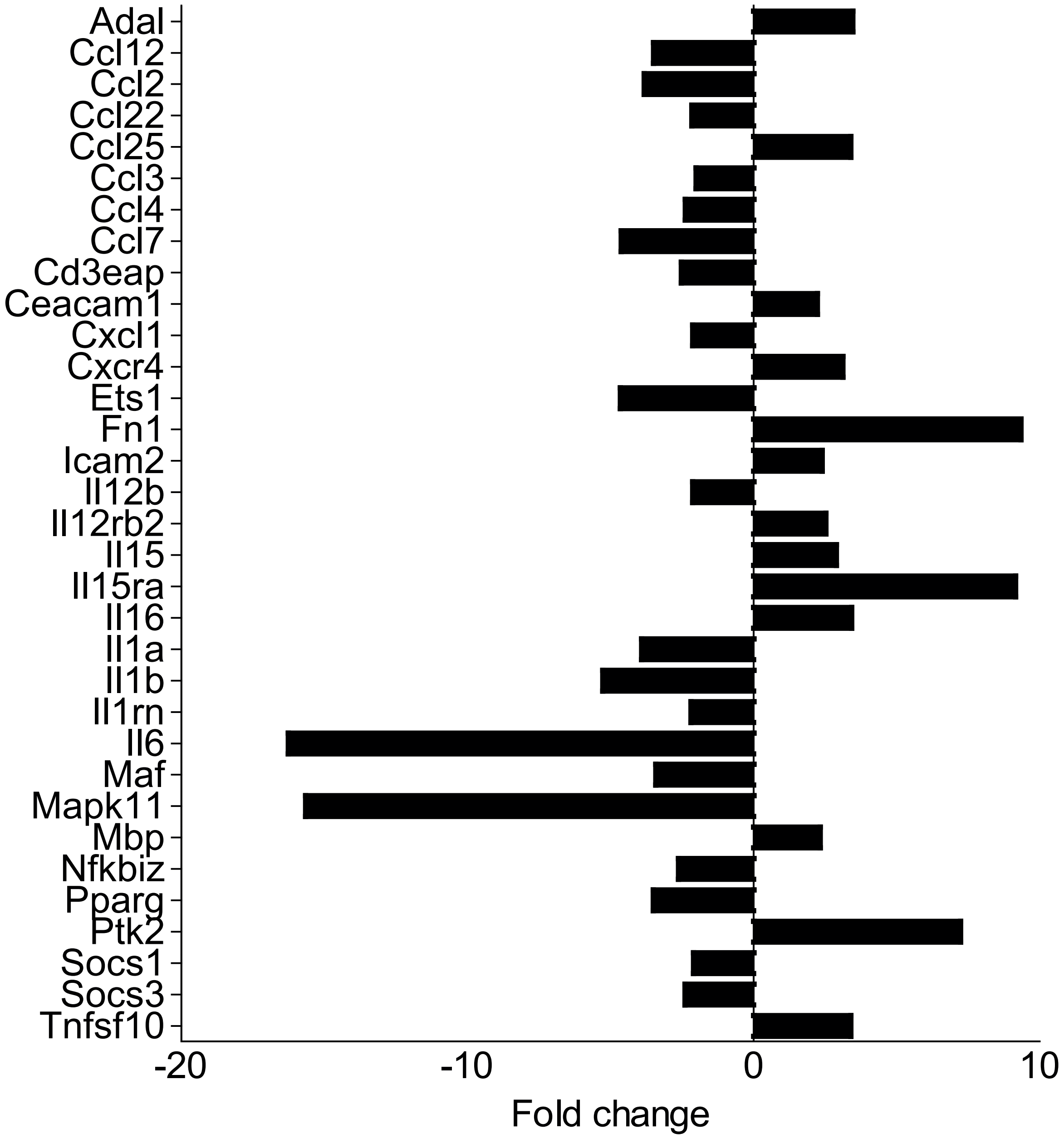

FIG S3 Nanostring data. Murine BMDMs were infected with the first and last clinical isolate of Mav (9.1 and 9.6) at a MOI of 10. After 6 hours post infection, cells were lysed in RLT buffer and subsequently RNA was extracted to perform nanostring analysis. nCounter® GX Mouse Inflammation Kit was used and the results were analyzed on *Partek® Genomics Suite®* software.

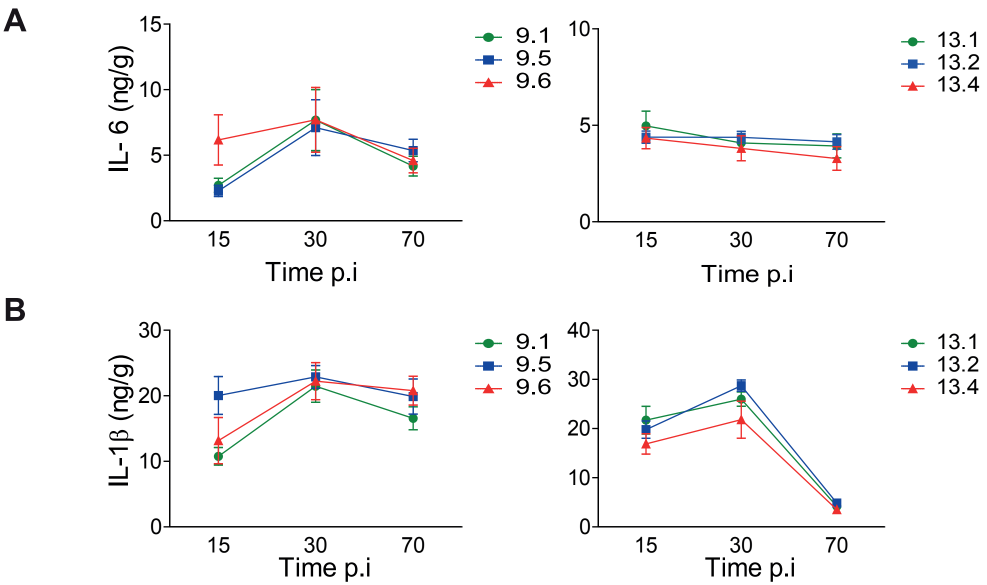

FIG S4 Proinflammatory cytokines IL-6 (A) and IL-1β (B) measured from liver homogenates.

Results are presented as ng cytokine per gram tissue.

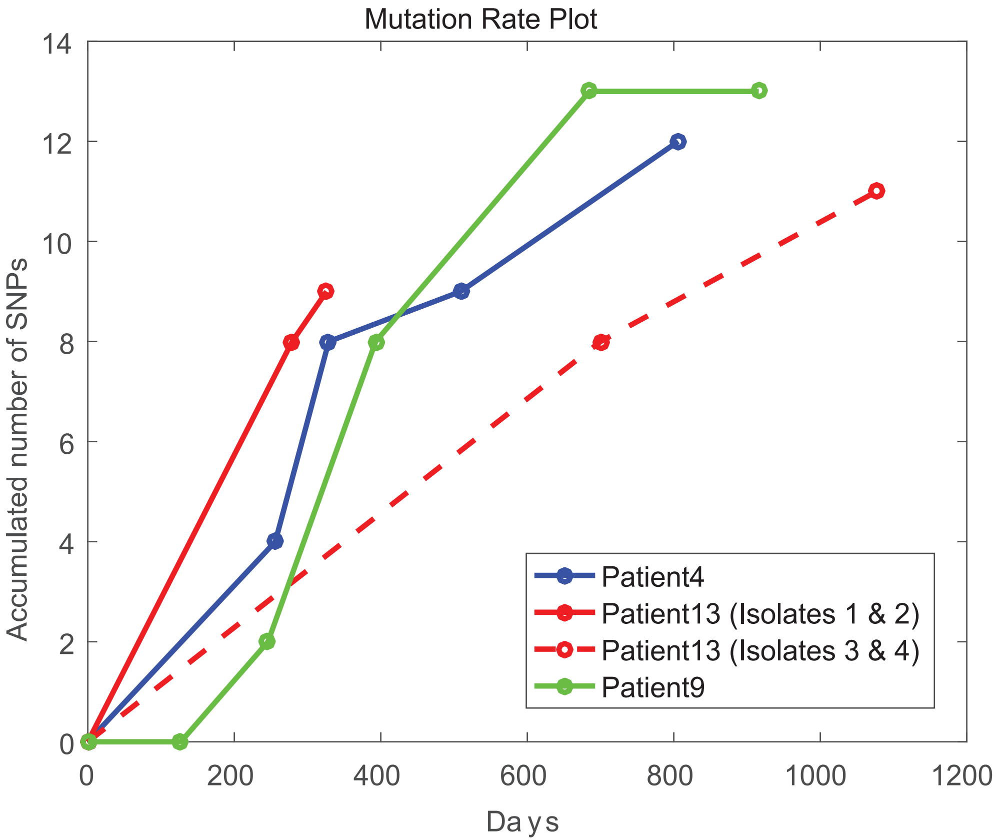

FIG S5 Accumulated number of SNPs, accounting for sub clones. SNPs were counted relative to Mav 104 for patients 9 and 13. The isolates from patient 13 were treated as two separate lineages (13.1-2 and 13.3-4). For patient 4, SNPs in isolates 4.2-4.6 were counted relative to 4.2.

Supplementary material and method

Melting curve assay

Quantitative Real-time Polymerase Chain Reaction (qRT-PCR) with melting curve analysis was performed on the patient isolates to confirm species identity, using Light Cycler® Fast Start DNA Master HybProbe Kit (Roche). DNA extraction was performed, using a NucliSENS® easyMAG® automated system (bioMerieux Clinical Diagnostics). The primers used to amplify the 16S rRNA gene region were MycoF: 5’-ACGGAAAGGTCTCTTCG-3’ and MycoR: 5’- GTCGTCGCCTTGGTAG-3’. The fluorogenic hybridization probes used were Myco Probe1: 5’-ATGTCTTGTGGTGGAAAGCG-fluorescein isothiocyanate-3’ and Myco Probe2: 5’-LC Red 640-TTAGCGGTGTGGGATGAGCCC-phosphate-3’. Mtb *H37Rv*, Mav 104 and *M. intracellulare* were used as mycobacterial reference strains.

Phylogenetic Analysis

A multiple alignment of the genome sequences from clinical strains and reference strain Mav 104 (NC_008595.1) and H87 was generated using MUMmer 3.23 (1)**.** SNPs were extracted from the multiple using a script (filtering out heterogeneous and low-coverage sites, as above). Sites involving indels in any of the isolates were also excluded. This resulted in a set of 12,560 polymorphic sites genome-wide. A phylogenetic tree was constructed by the maximum parsimony method (*dnapars*) in Phylip 3.66 (2)**.**

Identification of Single Nucleotide Polymorphisms (SNPs)

SNPs in the isolates from each patient were identified by aligning the assembled genome sequence from the first isolate to the sequences from isolates from each subsequent time point. SNPs resulting from heterogeneous nucleotide calls (<70% pure) or low-coverage regions (depth < 10) were filtered out.

Estimation of in vivo Mutation Rate

The apparent *in vivo* mutation rate of Mav was estimated by calculating the accumulated number of SNPs observed in each patient (compared to the first isolate) at each subsequent time point, divided by the time elapsed (in fractions of a year). For the second analysis, each isolate was compared to Mav 104. The 4-6 observations for each patient were fit to a Poisson distribution to derive the maximum likelihood estimate of the mean rate of accumulation of SNPs in each patient, along with 95% confidence intervals for the parameter estimates, using *poissfit* (rate_day*365) in MATLab.

Nanostring screen

Murine BMDMs were infected with the first and last clinical isolate of patient 9 (9.1 and 9.6) at a MOI of 10, for 6 hours. Cells were lysed in RLT buffer and subsequently RNA was extracted (Qiagen) to perform nanostring analysis. nCounter® GX Mouse Inflammation Kit (Nanostring technologies) was used to perform nanostring analysis on 100ng of RNA on a nCounter® digital analyzer (Nanostring technologies). Subsequently, values were imported into *Partek*® *Genomics Suite*® software for analysis where, 20 subtracted from normalized number of mRNA molecules, and set lowest value to 1, thereafter values were log_2-transformed and two-way

ANOVA was performed. Genes with a two-fold or higher (induced or repressed) change in expression level in macrophages infected with the last isolate compared to the first isolate were represented.

Cytokine quantification from infected macrophages

Macrophages were seeded at 200,000 cells/well in a 24 well plate and infected with Mav clinical isolates at a MOI of 10 for two hours before cells were washed to eliminate extracellular bacteria. Supernatants were harvested at 2h, 6h, 1, 3 and 7 days. IL-6 and IL-1β ELISAs (both from R&D systems) were performed on the supernatants according to the manufacturer's protocol.

Reference

1. Kurtz S, Phillippy A, Delcher AL, Smoot M, Shumway M, Antonescu C, Salzberg SL. 2004. Versatile and open software for comparing large genomes. Genome Biol 5:R12.

2. Felsenstein J. 1989. PHYLIP - Phylogeny Inference Package. Cladistics 5:163–166.

Table S1 Medical history and time line of sample collection for all the patients in the study**.**

Patient	Sex-age	Clinical history	First isolate	Last isolate	Interpretation	Treatment (start date)
**1**	F-50	Infiltrate right lung top in 2004, [i]COPD grade I	22.03.2006	01.06.2007	Asymptomatic colonization	No
**2**	M- 1 month	Lymphadopathy right side of neck	29.12.2008	09.02.2009	Infection	No, surgical removal.
**3**	F-57	Lung abscess in 1963, smoker, COPD grade 3-4, multiple cavernae and infiltrates, insulin dependent diabetes type II	10.08.2007	26.10.2010	First considered asymptomatic colonization, later as infection.	Rifampicin, myambutol and clarithromycin
**4**	M-65	Smoker, COPD, bulla and cavernae right lung top	23.03.2007	01.07.2010	Infection	First rifampicin, isoniazid and pyrazinamide, then ethambutol and clarithromycin. Treatment stopped due to resistance to all drugs
**5**	F-61	COPD/ emphysema grade 4, recurrent chest infections	23.05.2003	05.01.2005	Asymptomatic colonization	No
**7**	M-55	Colonic cancer diagnosed in 2003, chemotherapy, metastases to lung, chronic bronchitis?	07.12.2005	25.05.2007	Infection	Rifabutin, clarithromycin and ethambutol
**8**	F-71	Lung infiltrate with necrosis with granulomatous inflammation	22.05.2008	25.05.2008	Uncertain	Regress of infiltrate without antimycobacterial treatment
**9**	F-65	Bilateral lung infiltrates with bronchiectasis, cavernae and fibrosis	07.04.2005	10.10.2007	Infection	Rimactan and ethambutol
**10**	M-77	Severe bilateral lung fibrosis, MAC since 1990	24.05.2005	21.09.2007	Colonization	No

**11**	F-85	Pneumonia, lung infiltrate	07.07.2009	22.09.2010	Colonization	No, regression of infiltrate without antimycobacterial treatment
**12**	M-61	COPD/emphysema grade 4, caverna left lung top, infiltrate right midfield	09.04.2006	28.02.2007	Colonization	No,
**13**	F-83	Bronchiectasis, infiltrates and cavernae in both lung tops	20.12.2006	25.02.2009	Infection	No (risk of side effects, and patient not motivated for treatment)
**15**	F-55	Mild obstructive and restrictive lung disease, recurrent pneumonia, midfield infiltrate	19.01.2006	19.12.2006	Colonization	No, regress of infiltrate without antimycobacterial treatment

[i] Chronic Obstructive Pulmonary Disease (COPD)

Table S2 List of genes containing SNPs. Both synonymous and non-synonymous mutations were observed. Putative gene function mentioned for non-synonymous mutations. Mav 104 reference from KEGG database was used to annotate function. Mutations marked in red occur in or upstream of genes putatively related to drug resistance.

	**4.1**	**4.2**	**4.3**	**4.4**	**4.5**	**4.6**	**Annotations for Non-synonymous Mutations**
**Days**	0	390	646	719	899	1196	
***nc228631**						g>t	-57 bp upstream of *embB* (pseudogene)
**MAV_0570**			V62I	V62I	V62I	V62I	
**MAV_0809**			L256L	L256L	L256L	L256L	
**MAV_1067**		A128S	A128S	A128S	A128S	A128S	Glutmate synthetase
**MAV_1092**						+g (ins)	
**MAV_1302**			R27R	R27R	R27R	R27R	
**MAV_1393**						Y243C	
**nc1491618**				a>c	a>c	a>c	16S rRNA
**MAV_1537**				Q366E			AraC transcriptional regulator
**MAV_1658**				transposon ins	transposon ins	transposon ins	ortholog of Eis (aminoglycoside acetyltransferase)
**MAV_2013**		H1206Y	H1206Y	H1206Y	H1206Y	H1206Y	Mycobactin peptide synthetase MbtE
**MAV_2717**				T170A			Thiamine biosynthesis/ tRNA modification protein ThiI
**MAV_3418**			R493R	R493R	R493R	R493R	
**MAV_3565**		R221R	R221R	R221R	R221R	R221R	
**MAV_3604**		E51D	E51D	E51D	E51D	E51D	
**MAV_3626**		A136V	A136V	A136V	A136V	A136V	
**MAV_4065**		cluster of 4 SNPs	cluster of 4 SNPs	cluster of 4 SNPs	cluster of 4 SNPs	cluster of 4 SNPs	CheR methyltransferase
**MAV_4503**		E254Q					RpoB; DNA-

						directed RNA polymerase, beta subunit
**MAV_4827**	T330T	T330T	T330T	T330T	T330T	
**MAV_4838**	A289T	A289T	A289T	A289T	A289T	
**MAV_5303**				G205S		putative N-acetylmuramoyl -L-alanine amidase

* 'nc' means SNP occurs in a non-coding (or intergenic) region. The coordinate given is based on an alignment to the genome sequence of *M. avium* 104.

	**9.1**	**9.2**	**9.3**	**9.4**	**9.5**	**9.6**	**Annotations for Non-synonymous Mutations**
**Days**	0	127	246	393	685	916	
**MAV_0211**			A42V				
**MAV_5106**			Q525K				gamma-HCH transport system permease protein
**MAV_0166**				A1230T			ferredoxin-dependent glutamate synthase 1
**MAV_2082**				-c			ABC transporter
**MAV_2786**				A178P			phosphatidylethanolamine-binding protein
**MAV_3145**				E348D			exinuclease subunit B
**nc4424251**				g>t			in 20kb insertion in MAV_4292
**MAV_4983**				G33V			molybdopterin oxidoreductase
**MAV_0141**					I161L	I161L	nitrate/sulfate/bicarbonate transporter
**nc223899**					t>c	t>c	*embB* (pseudogene)
**nc1488761**					g>t		16S rRNA
**MAV_3262**					C175*		glycosyl transferase
**nc4439295**					c>t		near MAV_4309

	13.1	13.2	13.3	13.4	Annotations for Non-synonymous Mutations
**Days**	0	47	421	798	
**MAV_0030**			L4L	L4L	
**MAV_0048**				A488E	conserved hypothetical protein
**MAV_0182**				K38T	superoxide dismutase, Fe-Mn family
**MAV_0382**			P452A	P452A	
**MAV_0650**			A297S	A297S	
**MAV_1043**			E173E	E173E	
**MAV_2305**			L206L	L206L	
**MAV_2316**			S324P	S324P	conserved hypothetical protein
**MAV_2756**			Y150H	Y150H	methyltransferase
**MAV_2838**		F267V			homologue to the OxyR transcriptional regulator
**MAV_2876**			+agtg	+agtg	
**MAV_2967**			L146S	L146S	transcriptional regulator, TetR family protein
**MAV_3155**			M28K		DNA polymerase I
**MAV_3749**		K323E	K323E	K323E	DNA processing protein; smf family protein
**MAV_4147**			L239L	L239L	
**MAV_4190**			P144T	P144T	
**MAV_4900**			R289R	R289R	
**MAV_4964**				S239P	conserved hypothetical protein
**nc5110983**			t>c		near MAV_4966
**MAV_5255**			D46D	D46D	

.

Table S3 List of genes containing mutations (relative to Mav104, as an outgroup). Both synonymous and non-synonymous mutations were observed. Putative gene function mentioned for non-synonymous mutations. *M. avium* 104 reference from KEGG database was used to annotate function. The mutations for patient 13 are highlighted in red and blue to emphasize the two sub-series of isolates, (13.1, and 13.2) and (13.3, and13.4). The mutations for patient 4 are highlighted in red and blue to emphasize the two sub-series of isolates (4.1) and (4.2-4.6**).** Mutations in putative drug-resistance genes have been removed.

	4.1	4.2	4.3	4.4	4.5	4.6	Annotations for Non-synonymous Mutations
**Days (from 4.2)**	NA	0	256	329	509	806	
**MAV_0570**			V62I	V62I	V62I	V62I	
**MAV_0809**			L256L	L256L	L256L	L256L	
**MAV_1067**		A128S	A128S	A128S	A128S	A128S	Glutmate synthetase
**MAV_1092**						+g (ins)	
**MAV_1302**			R27R	R27R	R27R	R27R	
**MAV_1393**						Y243C	
**MAV_1537**				Q366E			AraC transcriptional regulator
**MAV_2013**		H1206Y	H1206Y	H1206Y	H1206Y	H1206Y	Mycobactin peptide synthetase MbtE
**MAV_2717**				T170A			Thiamine biosynthesis/ tRNA modification protein ThiI
**MAV_3418**			R493R	R493R	R493R	R493R	
**MAV_3565**	R221R						
**MAV_3604**	D51E						
**MAV_3626**		A136V	A136V	A136V	A136V	A136V	
**MAV_4065**	cluster of 4 SNPs						CheR methyltransferase
**MAV_4827**	T330T						
**MAV_4838**		A289T	A289T	A289T	A289T	A289T	
**MAV_5303**					G205S		putative N-acetylmuramoyl -L-alanine

							amidase

* 'nc' means SNP occurs in a non-coding (or intergenic) region. The coordinate given is based on an

	**9.1**	**9.2**	**9.3**	**9.4**	**9.5**	**9.6**	**Annotations for Non-synonymous Mutations**
**Days (from 9.1)**	0	127	246	393	685	916	
**MAV_0211**			A42V				
**MAV_5106**			Q525K				gamma-HCH transport system permease protein
**MAV_0166**				A1230T			ferredoxin-dependent glutamate synthase 1
**MAV_2082**				-c			ABC transporter
**MAV_2786**				A178P			phosphatidylethanolamine-binding protein
**MAV_3145**				E348D			exinuclease subunit B
**nc4424251**				g>t			
**MAV_4983**				G33V			molybdopterin oxidoreductase
**MAV_0141**					I161L	I161L	nitrate/sulfate/bicarbonate transporter
**MAV_3262**					C175*		glycosyl transferase
**nc4439295**					c>t		

alignment to the genome sequence of *M. avium* 104.

	13.1	13.2	13.3	13.4	Annotations for Non-synonymous Mutations
**Days (from first diagnosis*)**	279	326	700	1077	
**MAV_0030**	L4L	L4L			
**MAV_0048**				A488E	conserved hypothetical protein
**MAV_0182**				K38T	superoxide dismutase, Fe-Mn family
**MAV_0382**			P452A	P452A	
**MAV_0650**	S297A	S297A			
**MAV_1043**	E173E	E173E			
**MAV_2305**			L206L	L206L	
**MAV_2316**			S324P	S324P	conserved hypothetical protein
**MAV_2756**			Y150H	Y150H	methyltransferase
**MAV_2876**	-agtg	-agtg			
**MAV_2967**			L146S	L146S	transcriptional regulator, TetR family protein
**MAV_3155**			M28K		DNA polymerase I
**MAV_3749**	E323K				DNA processing protein; smf family protein
**MAV_4147**	L239L	L239L			
**MAV_4190**	T144P	T144P			
**MAV_4900**			R289R	R289R	
**MAV_4964**				S239P	conserved hypothetical protein
**nc5110983**			t>c		
**MAV_5255**	D46D	D46D			

* For patient 13, 279 days from the first diagnosis was added as an estimate of the time in-patient during which mutations accumulated, assuming that the two lineages accumulated distinct sets of mutations from a common progenitor during the course of infection.

Table S4 Description of mycobacterial isolates; Specimen type, Specimen location, microscopy quantification and concentration of bacteria

Patient ID	Isolate ID	Specimen received date	Specimen type	Specimen location	Microscopy	Concentration of Bacteria
1	1-1	22/03/2006	BAL	lung	AFB not detected	low
1	1-2	1/06/2007	BAL		AFB not detected	low
2	2-1	29/12/2008	Abscess	Neck	Not done	Uncertain
2	2-2	9/02/2009	Tissue biopsy	Neck	AFB detected, not quantified	Uncertain
3	14-1	10/08/2007	BAL	Lung	AFB not detected	low
3	14-2	19/12/2008	Sputum	Lung	>9 AFB/field	high
3	3-1	26/01/2010	BAL	Lung	>9 AFB/field	high
3	3-2	5/08/2010	Sputum	Lung	Not done	Uncertain
3	3-3	26/10/2010	Sputum	Lung	AFB detected, not quantified	Uncertain
4	6-1	23/03/2007	Sputum	Lung	>9 AFB/field	high
4	6-2	16/04/2008	Sputum	Lung	10 AFB/field	high
4	6-3	28/12/2008	Sputum	Lung	>9 AFB/field	high
4	4-1	11/03/2009	Sputum	Lung	>9 AFB/field	high
4	4-2	7/09/2009	Sputum	Lung	>9 AFB/field	high
4	4-3	1/07/2010	BAL	Lung	AFB not detected	low
5	5-1	23/05/2003	Sputum	Lung	AFB not detected	low
5	5-2	5/01/2005	BAL	Lung	AFB not detected	low

7	7-1	7/12/2005	Bal	Lung	2 AFB/300 fields	high
7	7-2	25/05/2007	Transbronchial biopsy	Lung	AFB not detected	low
8	8-1	22/05/2008	Sputum	Lung	>9 AFB/field	high
8	8-2	26/05/2008	Transbronchial biopsy	Lung	AFB detected, not quantified	high
9	9-1	7/04/2005	Sputum	Lung	1 AFB/field	high
9	9-2	12/08/2005	Sputum	Lung	5 AFB/100 fields	high
9	9-3	9/12/2005	Sputum	Lung	5 AFB/field	high
9	9-4	5/05/2006	Sputum	Lung	>9 AFB/field	high
9	9-5	21/02/2007	Sputum	Lung	>9 AFB/field	high
9	9-6	10/10/2007	Sputum	Lung	1 AFB/5 fields	high
10	10-1	25/04/2005	Sputum	Lung	3 AFB/10 fields	high
10	10-2	11/06/2007	Sputum	Lung	AFB not detected	low
10	10-3	21/09/2007	Tissue?	Not specified, probably transbrochial	AFB not detected	low
11	11-1	7/07/2009	BAL	Lung	AFB not detected	low
11	11-2	22/09/2010	Sputum	Lung	AFB not detected	low
12	12-1	9/04/2006	Sputum	Lung	>9 AFB/field	high
12	12-2	28/02/2007	Sputum	Lung	>9 AFB/field	high
13	13-1	20/12/2006	Sputum	Lung	AFB not detected	low
13	13-2	5/02/2007	Sputum	Lung	AFB not detected	low
13	13-3	14/02/2008	BAL	Lung	AFB not detected	low
13	13-4	25/02/2009	Sputum	Lung	6 AFB/field	high
15	15-1	19/01/2006	BAL	Lung	AFB not detected	low
15	15-2	19/12/2006	Sputum	Lung	AFB not detected	low

Table S5. MAV genes potentially related to drug resistance. This list was generated by identifying orthologs in the *M. avium* 104 genome of genes related to drug resistance in *M. tuberculosis* for drugs commonly used to treat mycobacterial infections (1).

MAV_0005	Rv0005	*gyrB*
MAV_0006	Rv0006	*gyrA*
MAV_0173	Rv3855	*ethR*
MAV_0175	Rv3854c	*ethA*
MAV_0225	Rv3794	*embA*
MAV_0224	Rv3795	*embB*
MAV_0229	Rv3793	*embC*
MAV_0595	Rv3566c	*nat*
MAV_1418	Rv1267c	*embR*
MAV_1658	Rv2416c	*eis*
MAV_1725	Rv2447c	*folC*
MAV_2190	Rv2247	*accD6*
MAV_2192	Rv2245	*kasA*
MAV_2194	Rv2243	*fabD*
MAV_2472	Rv2043c	*pncA*
MAV_2752	Rv1909c	*furA*
MAV_2753	Rv1908c	*katG*
MAV_2839	Rv2428	*ahpC*
MAV_2867	Rv1854c	*ndh*
MAV_2944	Rv1772	-
MAV_2992	Rv0678	-
MAV_3077	Rv1694	*tlyA*
MAV_3152	Rv1630	*rpsA*
MAV_3294	Rv1484	*inhA*
MAV_3295	Rv1483	*fabG1*
MAV_3652	Rv2764c	*thyA*
MAV_3702	Rv2846c	*efpA*
MAV_4018	Rv3139	*fadE24*
MAV_4231	Rv3266c	*rmlD*
MAV_4471	Rv0701	*rplC*
MAV_4492	Rv0682	*rpsL*
MAV_4503	Rv0667	*rpoB*
MAV_4664	Rv0486	*mshA*
MAV_5183	Rv0129c	*fbpC*
MAV_5308	Rv3919c	*gid*
MAV_2838	Rv2427A	*oxyR*
5 kb region around coord 1.49 Mb	Rvnr01	*rrs*

1. Mortimer TD, Weber AM, Pepperell CS. 2018. Signatures of Selection at Drug Resistance Loci in Mycobacterium tuberculosis. mSystems 3.

Table S6 : Primers for Sanger Sequencing

Gene	Primer	Sequence
Pat4_MAV_1067	Forward	5’AAGCTCGGCTCCGGCAAACTG3’
Pat4_MAV_1067	Reverse	5’CACCCAGCGAGCTCAGGC3’
Pat4_MAV_1537	Forward	5’CGTTTGGTCGCCGCCTAC 3’
Pat4_MAV_1537	Reverse	5’GCAATTGTGTTCGGGCGC 3’
Pat4_MAV_2717	Forward	5’ GACCTGGGGTGGCCGGTC 3’
Pat4_MAV_2717	Reverse	5’ ATCGCAGGCCAGTCCGCG 3’
Pat4_MAV_5303	Forward	5’GTCCGCCGATCGGGCCCC 3’
Pat4_MAV_5303	Reverse	5’CAGCGGGCTGCGGTTGACC 3’
Pat9_MAV_0141	Forward	5’ CCGTACTTCTTCCTGTTGC 3’
Pat9_MAV_0141	Reverse	5’CAGCACACCGCCGGAGTT 3’
Pat9_MAV_0166	Forward	5’ GTGAAGTCGCTGGACACCAC 3’
Pat9_MAV_0166	Reverse	5’ AGAACCGCACCGGCTTGCC 3’
Pat9_MAV_2786-F	Forward	5’GGCTCCGGCAGCACGTCC 3’
Pat9_MAV_2786-R	Reverse	5’ CGCGGCGGCGCCGATCGT 3’
Pat13_MAV_0182	Forward	5’GTGGCTGAATACACCCTG 3’
Pat13_MAV_0182	Reverse	5’CAGGTGGAAGGCGAGGTT3’
Pat13_MAV_2756	Forward	5’AGCAACGAAGGCGGCCACG 3’
Pat13_MAV_2756	Reverse	5’CTGGGACCTGATGCGGCGA 3’
Pat13_MAV_3155	Forward	5’GTGCCCGCCACGAAAGCCG 3’
Pat13_MAV_3155	Reverse	5’CAGCAGGTTGATCAGCATCGC3’
Pat13_MAV_3749	Forward	5’ATCGGCCACATCGGCGAATTGG 3’
Pat13_MAV_3749	Reverse	5’ CTGGCGGCGCACCAGCCC 3’
